# Harnessing Anthocyanins to Mitigate Inflammation, Dysbiosis, and Aging in the Gastrointestinal Tract

**DOI:** 10.1021/acsptsci.5c00566

**Published:** 2026-01-28

**Authors:** Livia Resende Lopes, Adriel Aparecido de Souza, Tanila Wood dos Santos, Raquel de Cássia dos Santos

**Affiliations:** Post Graduate Program in Health Sciences, Laboratory of Natural Products, São Francisco University, 12916-900 Bragança Paulista, São Paulo, Brazil

**Keywords:** anthocyanins, gut microbiota, cellular senescence, immune homeostasis, inflammatory bowel disease (IBD)

## Abstract

The gut microbiota are a dynamic ecosystem that is crucial for immune regulation and maintenance of intestinal barrier integrity. Dysbiosis within this community contributes to the chronic inflammation characteristic of inflammatory bowel diseases (IBD), including Crohn’s disease and ulcerative colitis, for which no definitive cure currently exists. This comprehensive review examines recent preclinical and clinical studies on how anthocyanin-polyphenolic pigments, such as cyanidins and malvidins, modulate gut microbial communities, reduce intestinal inflammation, and counteract age-related declines in immune homeostasis. We analyzed the literature on anthocyanin–microbiota interactions in IBD pathogenesis, focusing on cytokine profiles, barrier function assays, lipopolysaccharide synthesis, oxidative stress markers, and short-chain fatty acid production. Additionally, we explored the relationship among cellular senescence, the senescence-associated secretory phenotype (SASP), and microbiome shifts during intestinal aging. Evidence indicates that anthocyanins consistently suppress key pro-inflammatory cytokines, such as interleukin-1β, interleukin-6, TNF-α, and interferon-γ, while preserving mucosal architecture and reducing lipopolysaccharide load and mitochondrial oxidative phosphorylation. These compounds help to restore microbial balance, promote short-chain fatty acid synthesis, and enrich bacterial taxa associated with barrier integrity. In aging models, anthocyanins attenuate oxidative stress, stabilize redox homeostasis, inhibit senescence signaling and SASP secretion, and partially restore anti-inflammatory interleukin-10 levels. In conclusion, anthocyanins are promising dietary therapeutics for IBD management and for mitigating intestinal aging. Future research should transition from murine models to human clinical trials by integrating senolytic strategies, targeted microbiome modulation, and pharmacological dissection of the senescence–microbiome axis to foster disease prevention and promote healthy aging.

## Graphical abstract

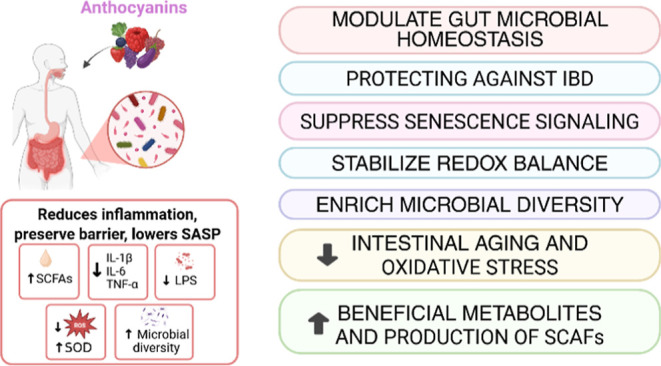

## Introduction

Healthy human microbiomes are composed of many microorganisms, such as bacteria, viruses, and yeasts, which live on the skin, mouth, gastrointestinal, respiratory, and genitourinary tracts. These microorganisms perform several important functions such as aiding in the digestion and absorption of nutrients and promoting proper functioning of the immune and nervous systems. In particular, the intestinal microbiome contains genes encoding enzymes and metabolites that aid in digestion. In contrast, the gastrointestinal microbiome is an interface between the external environment, food, and the human body. Factors such as age, diet, and antibiotic use can affect the diversity of the gastrointestinal microbiome, which may be linked to diseases such as colorectal cancer, inflammatory bowel disease, irritable bowel syndrome, and diabetes.^1,2^ The first source of intestinal microorganisms occurs during childbirth through direct contact with the mother’s fecal microbiota, followed by the environment and breastfeeding.^3^ After birth and up to three years of age, microbiota develops, modifying the prevalent microorganisms.^4^ However, Perez-Muñoz^5^ indicated that the first colonization occurred in the uterus of the mother, without any evidence of rupture of the amniotic barrier. According to Aagaard et al.,^6^ the microbiota present in the placenta comprises commensal microorganisms belonging to the following phyla: *Tenericutes*, *Firmicutes*, *Bacteroidetes*, *Proteobacteria,* and *Fusobacteria*. In relation to amniotic fluid, the microbial community is distinct in amniotic fluid; however, it is characterized by low diversity, low richness, and a predominance of *Proteobacteria*. An important point is that in the first meconium, there is already a complex microbiota, with *Prevotella* being the predominant species.

Diversification of the intestinal microbiome, as shown in Figure 1, is due to several factors, such as the type of delivery. Normal deliveries have a predominance of *Prevotella* spp. and *Lactobacillus* spp. since they are exposed to the maternal vagina and the mother’s fecal microbiota. However, the microbiome of those born by cesarean section is dominated by microorganisms derived from the mother’s skin, environment, and hospital staff, including *Corynebacterium*, *Staphylococcus,* and *Propionibacterium* spp.^8^ Babies born by cesarean section have a greater number of species of bacteria that colonize the skin and hospital environment; there is an increased chance of acquiring an infection by an opportunistic pathogen, such as *Klebsiella pneumoniae*.^3^ Furthermore, according to Kalbermatter,^3^ babies born by surgical delivery have 30% fewer bacterial species shared with their mothers. Babies born by cesarean section have a greater number of bacterial species that colonize their skin and the hospital environment, and there is an increased chance of acquiring an infection by an opportunistic pathogen such as *K. pneumoniae*. In addition, babies born by surgical delivery have 30% fewer bacterial species than their mothers. It is also worth noting that, in the first week after birth, the predominance of bacteria belongs mainly to the phyla *Actinobacteria* and *Bacteroidetes* in vaginal newborns and *Firmicutes* in newborns born via cesarean section.^9^ Different genera of bacteria have important biological functions in intestinal microbiota (Figure 2).

**Figure 1:**
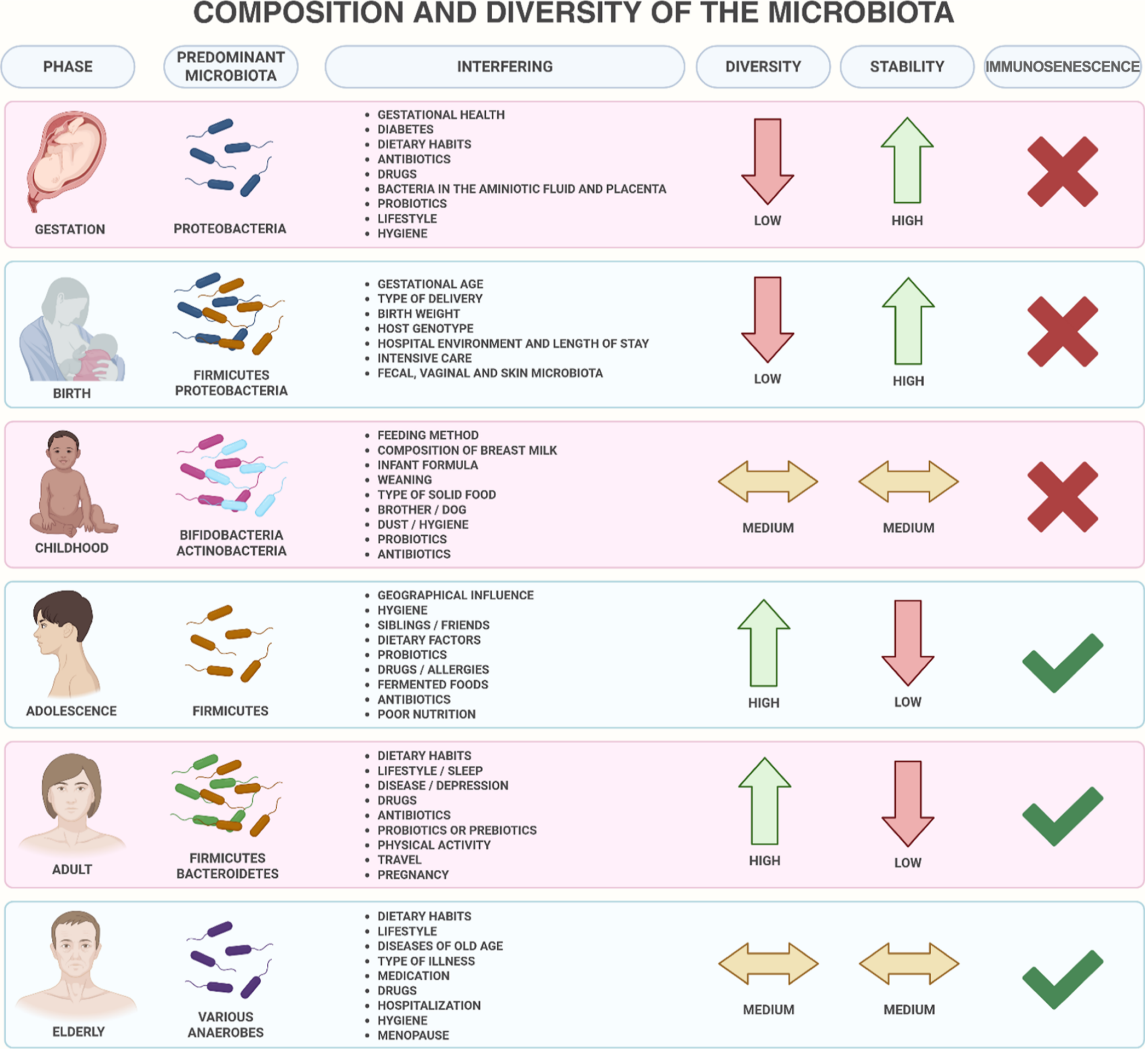
Age-related alterations in the human gut microbiome and key determinants influencing microbiota composition across the lifespan.^7^ Created in BioRender. Pereira, Q. (2025) https://BioRender.com/542v716.

**Figure 2:**
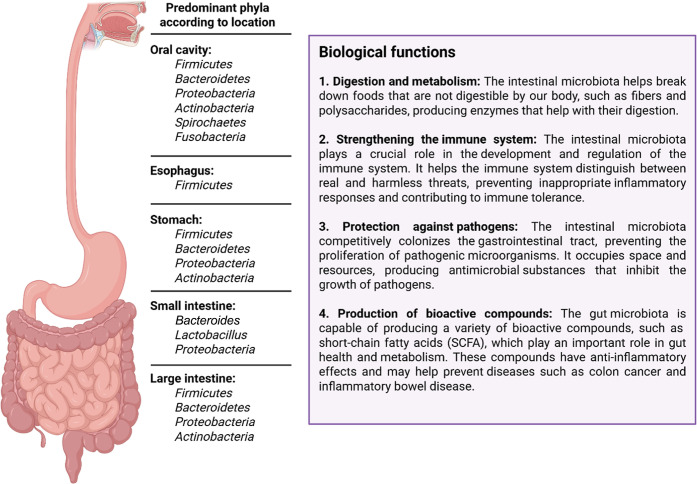
Spatial distribution of the predominant bacterial phyla along the gastrointestinal tract (oral cavity, esophagus, stomach, and intestine (small and large)), with the main biological functions associated with intestinal homeostasis.^1–3^ Created in BioRender. Pereira, Q. (2025) https://BioRender.com/542v716.

Microbial composition and density vary along the gastrointestinal tract in response to local secretions, gastric acid in the stomach, and pancreatic juice plus digestive enzymes in the duodenum, resulting in relatively low bacterial loads, which progressively increase throughout the small intestine and reach their highest concentrations in the colon.^10^ The term microbiota indicates the microorganisms present in a defined environment, such as the intestine, and the term dysbiosis indicates the disturbance in the balance of these microorganisms; that is, there is a change in the composition and relationship of the different phyla along the gastrointestinal tract.^11^ Dysbiosis describes the disruption of the normal composition of the gut microbiota and function, marked by phylum-level shifts, reduced microbial diversity, overgrowth of pathobionts, loss of beneficial commensals, and ectopic bacterial colonization. It is driven by factors such as poor diet, antibiotic misuse, physical inactivity, smoking, aging, and genetic predisposition. Dysbiosis has a bidirectional relationship with numerous disorders, including irritable bowel syndrome, IBD, type 2 diabetes, chronic liver disease, obesity, cardiovascular and kidney diseases, and neuropsychiatric conditions, both contributing to disease onset and exacerbating by the pathological state.^11,12^

Some of the possible therapeutic approaches for dysbiosis may be probiotics (microorganisms that bring benefits to the host, such as rebalancing the intestinal microbiota and reducing intestinal permeability), prebiotics (food components that stimulate the balance and restoration of the intestinal microbiota), and/or fecal microbiota transplantation (application of live microorganisms from donors in varying quantities aiming at eubiosis).^13,14^

Dysbiosis often coincides with increased intestinal permeability driven by the loss of protective bacteria or the overgrowth of pathogens. Mucosal inflammation further compromises the barrier function, creating a self-reinforcing cycle of microbial imbalance and epithelial disruption.^15^

### Intestinal Permeability and Diseases

Changes in the flow of solutes and fluids between the lumen and tissues across the epithelium are referred to as changes in intestinal permeability. The intestinal epithelia are supported by the cytoskeleton. They extend across the lateral-apical portions of cells and form tight junction (TJ) proteins, which are composed of membrane proteins, occludins, claudins, and cytoplasmic proteins that serve as access routes for macromolecules, which allow or do not allow the bidirectional passage of substances.^16^ Transcellular routes allow molecules smaller than 0.4 nm to cross cell membranes through small, high-incidence aqueous pores present in the enterocyte membrane. As for paracellular routes, molecules larger than 0.5 nm cross larger aqueous channels in TJ proteins, which are of low incidence and susceptible to hyperosmolar stress.^15^ Generally, macromolecule permeation increases during processes that cause inflammatory reactions in the intestinal mucosa. Furthermore, disruption of the intestinal barrier and increased macromolecule permeation have been associated with etiopathogenic mechanisms common to inflammatory diseases of the gastrointestinal tract and autoimmune diseases.^17^

Zonulin, an important biomarker of intestinal permeability, belongs to the prehaptoglobin 2 (HP2) protein family, which describes the pathogenic role of the leaky gut in a variety of chronic inflammatory diseases (CIDs). When there is a change in intestinal permeability, the bacteria present in the intestinal lumen can translocate to other regions and induce inflammation, consequently causing systemic damage to the tissue if translocated to the peripheral circulation. In addition, activation of the zonulin pathway is a physiological mechanism that maintains mucosal homeostasis. However, it is worth noting that this biomarker is not involved in all CIDs and not all CIDs are associated with increased intestinal permeability.^12,15^ Polyphenols have been used to reduce intestinal permeability and improve the function of TJ proteins by interfering with transduction pathways. However, adverse effects occur, such as pro-oxidant activity, disruption of transporters, and the modulation of some enzymes. Intestinal alkaline phosphatase is actively anchored to the epithelial membrane or secreted into the intestinal lumen; thus, it can increase the number of lipopolysaccharide-suppressing bacteria (*Bifidobacterium*) and reduce the number of lipopolysaccharide-producing bacteria (*Escherichia coli*).

The gastrointestinal mucosa is a barrier that allows and limits the passage of substances through its permeability and is regulated by TJ, which promotes the adhesion of epithelial cells to each other and seals the intercellular spaces. The interaction between them and the intestinal microbiota has been suggested as one of the main contributors to TJ remodeling, as the metabolites of bacteria present in the intestine can serve as regulators of this barrier.^18^

Figure 3 shows the differences between the intestinal barrier under conditions of homeostasis and imbalance, which makes the intestinal mucosa thinner, allowing increased intestinal permeability.

**Figure 3:**
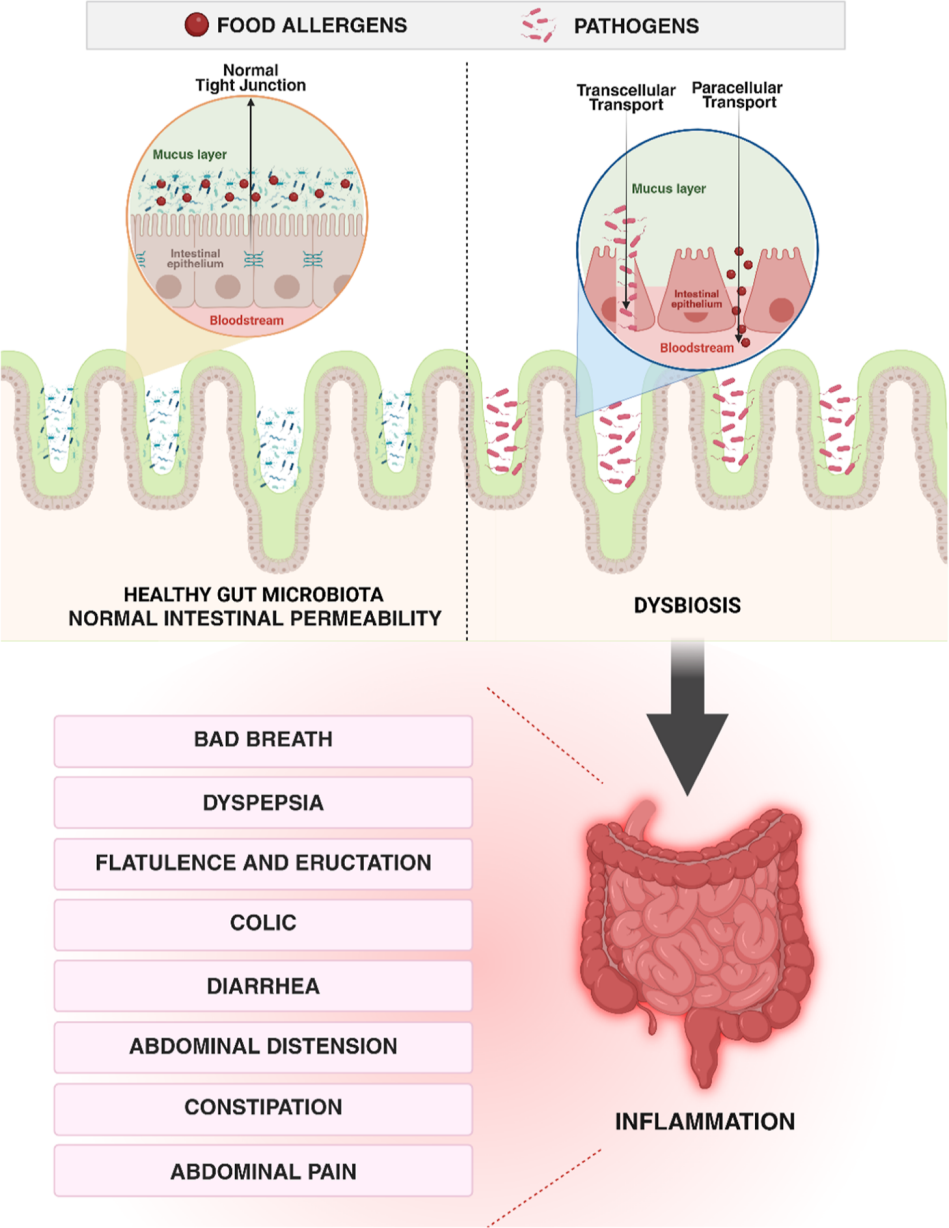
Comparison between normal intestinal epithelium in eubiosis and the epithelium under dysbiosis with increased permeability. Left panel (healthy eubiosis): intact epithelial monolayer with organized tight junctions and a continuous mucus layer, a diverse commensal microbiota, and balanced mucosal immunity resulting in selective permeability and normal digestive function. Right panel (dysbiosis and increased permeability): disrupted epithelial integrity with reduced or mislocalized tight junction proteins and a thinner mucus layer, loss of microbial diversity with expansion of pathobionts, heightened mucosal inflammation, and increased paracellular permeability leading to antigen translocation and clinical manifestations such as abdominal pain, bloating, and altered bowel habits.^11,19^ Created in BioRender. Pereira, Q. (2025) https://BioRender.com/542v716.

This results in the absorption of lipopolysaccharides (LPS), a component of the outer membrane of Gram-negative bacteria, along with food particles, into the systemic circulation, producing metabolic endotoxemia. The resulting chronic inflammatory state can contribute to inflammatory bowel disease (IBD), with its main representatives being CD and UC (Figure 4) occurs due to genetic predisposition but has mainly been associated with lifestyle, consumption of processed and ultraprocessed foods, greater exposure to pollutants, consumption of alcohol and drugs, and conditions related to the process of urbanization and industrialization, among other factors that impair intestinal homeostasis and generate immunological responses that lead to chronic intestinal inflammation, causing a significant impact on an individual’s life.^20,21^ The diagnosis of these diseases can be made through the association of clinical information with serological, radiological, endoscopic, and histological examinations.^22^

**Figure 4:**
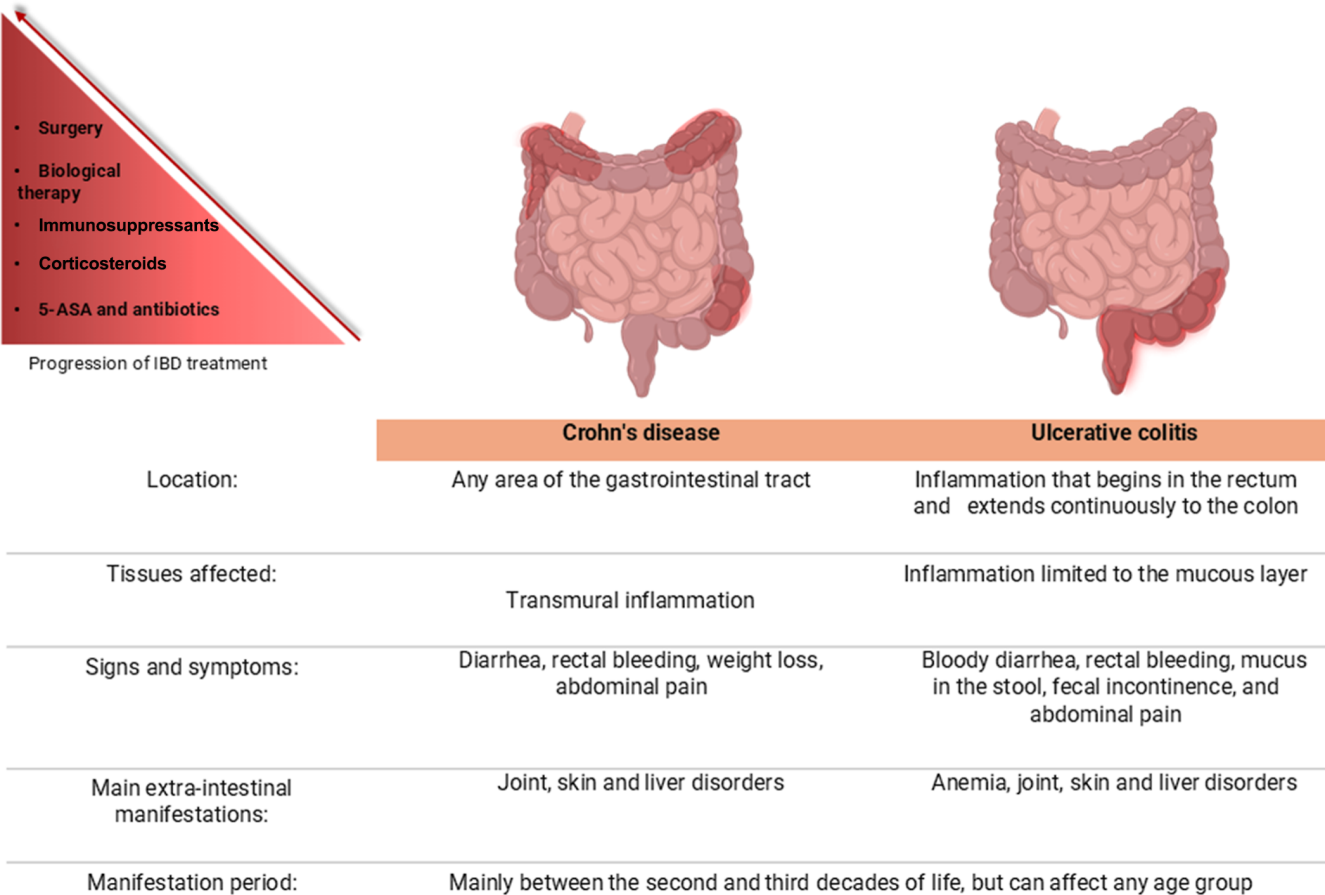
Most prevalent IBD and important differences^23–25^ and main classes of drugs and their representatives used in the treatment of IBD.^26^ Created in BioRender. Pereira, Q. (2025) https://BioRender.com/542v716.

Due to the scarcity of epidemiological studies in Brazil that quantify the incidence and prevalence rates of IBD and the under-reporting of these diseases, it is difficult to determine the exact number of affected individuals. However, in 2021, the Brazilian Society of Coloproctology reported that the prevalence of IBD varies from 12 to 55 per 100,000 inhabitants, with the increase in diagnoses being characteristic of developing countries, such as Brazil. Furthermore, between 2020 and 2021, in a multicenter study carried out in three states in Northeast Brazil, Pernambuco, Paraíba, and Rio Grande do Norte, a progressive increase in IBD diagnoses in the region was reported, with the behavior of the disease being described mainly as extensive, difficult to control, and frequently associated with complications, which may be partly due to the prolonged time between the onset of symptoms and the diagnosis of the disease.^27^ It is important to consider that IBD is not cured; however, it can be treated with drugs that allow achieving and maintaining remission of the disease and that the choice of treatment is guided by age, comorbidities, symptoms, state of inflammation, the location and extent of the disease, and the overall risk of more severe and complicated disease.^28^ The main classes of drugs used to treat IBD are salicylic derivatives (e.g., mesalazine and sulfasalazine), corticosteroids (e.g., budesonide, prednisone, and prednisolone), immunosuppressants (e.g., azathioprine, 6-mercaptopurine, methotrexate, and cyclosporine), and biological therapies (e.g., anti-TNF antibodies). Furthermore, when there is no clinical response to drug therapy, surgical intervention is possible^26^ (Figure 5). However, in addition to medications with several side effects, they do not promote complete remission, leading to a search for new molecules.

**Figure 5:**
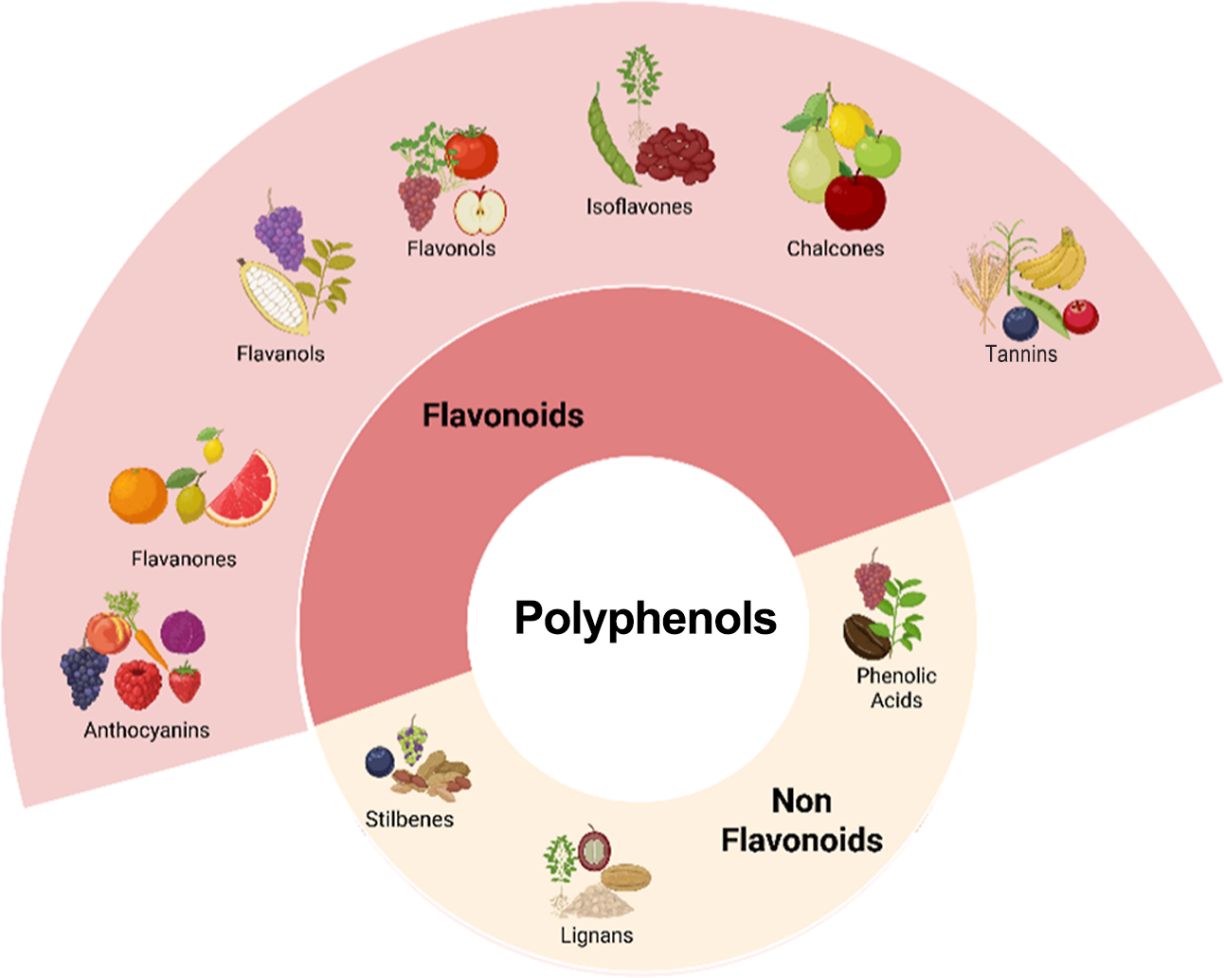
Overview of major classes of phenolic compounds found in plants with representative subgroups.^31,35^ Created in BioRender. Pereira, Q. (2025) https://BioRender.com/542v716.

Given the growing need and considering the recurrence of IBD, therapies with limitations and severe side effects, adjuvant diets, and natural products, the focus of this review was to analyze whether the anthocyanins cyanidin and malvidin, naturally present in foods, have therapeutic potential as well as the relationship of these substances with the intestinal microbiota and their possible pharmacological activities.

## Methodology

A literature search was performed in PubMed from August 2023 to November 2023. We used the following keywords and MeSH terms: “anthocyanins” (including subclasses cyanidin, malvidin, delphinidin, pelargonidin, peonidin, and petunidin), “gut microbiota”, “dysbiosis”, “inflammatory bowel disease”, “senescence”, and “aging”. Only the original research articles published in English between January 2018 and December 2024 were considered. Titles and abstracts were first screened for relevance, and full texts were assessed against predefined inclusion and exclusion criteria. We included studies in which anthocyanins were the primary bioactive compounds of interest and specifically evaluated their effects on gut microbial composition, intestinal inflammation, and aging-related parameters. Studies were excluded if they (1) presented incomplete or preliminary data, (2) did not focus primarily on anthocyanins despite reporting their presence in complex mixtures, or (3) were unavailable in the full text.

## Results and Discussion

### Anthocyanins and Intestinal Alterations

Dietary polyphenols, which are abundant in fruits, vegetables, and other plant-derived foods, exert protective effects against obesity, type 2 diabetes mellitus, cardiometabolic disorders, and neurodegenerative diseases largely through their antioxidant and anti-inflammatory properties. Accumulating evidence demonstrates that polyphenols reshape the colonic microbiota, promoting eubiosis by selectively stimulating commensal taxa and inhibiting pathobionts. These microbiota-mediated actions strengthen epithelial barrier integrity, suppress mucosal inflammatory signaling, and enhance mucosal immune defenses.^29–31^ Polyphenols, such as anthocyanins and flavonoids present in fruits and vegetables, are used to reduce intestinal permeability and improve TJ function, acting against chronic inflammatory processes. In addition, their beneficial effects on the intestine have been studied because the action of these substances may be related to other pathways, such as their interaction with the intestinal microbiota. The ingestion of anthocyanins stimulates the growth of beneficial bacteria in the intestine, such as *Lactobacillus* spp. and *Bifidobacterium* spp., thus favoring balanced and healthy microbiota.^32,33^ In addition, phenolic compounds shown in Figure 5, more precisely, those present in anthocyanins, can be used by intestinal bacteria as substrates to produce energy and fermentable metabolites.^34^

Anthocyanins are formed by an anthocyanidin (aglycone form); that is, they are the glycosidic form of anthocyanidins. Each aglycone can be glycosylated or acylated using different sugars and aromatic or aliphatic acids.^36^ They are considered secondary metabolites produced from biotic or abiotic stresses suffered by plants; thus, they act to protect plants against insect attacks, excess light, sudden temperature fluctuations, and nutritional deficiencies. Figure 5 demonstrates the role of anthocyanins as an important group of pigments responsible for the variation in colors, such as red, orange, purple, and blue, in flower petals and fruits.

It has been suggested that after ingestion, anthocyanins can be transported to the colon, where the intestinal microbiota actively produces anthocyanin metabolites.^37^

Malvidin is an organic compound derived from anthocyanins with methyl substituents at the 3′ and 5′ positions and is therefore considered a methylated anthocyanin. This substance is visible in purple and abundant in blue flowers. It is also the main red pigment in red wine^38^ and is present in fruits such as grapes and blueberries. In addition, it is highly soluble in water and can be metabolized to syringic acid. Among the main anthocyanin compounds, we identified the largest group of anthocyanin classes known as cyanidins. These compounds are natural pigments that provide color for various foods such as blueberries, cherries, grapes, and blackberries.^39^ In addition to providing vibrant colors, cyanidins have aroused scientific interest because of their potential benefits to human health (Figure 6). Petunidin, on the other hand, is an anthocyanidin similar to peonidin in terms of its chemical structure; however, it has methoxy (–OCH3) instead of hydroxyl groups. It is also found in various plants, such as grapes, blackberries, blueberries, and flowers, such as petunia. Petunidin imparts a deep purple color to plants, has antioxidant properties, and is beneficial to health.^40^ Peonidin has a specific chemical structure characterized by a benzene ring with two hydroxyl groups (–OH) and a chromophore structure. It can be found in a variety of plants and flowers, such as rose and peony.^41^ Peonidin is known for its bright red color and antioxidant and anti-inflammatory properties, which may be beneficial to human health.^42^ Pelargonidin is an organic compound with hydroxyl groups at positions 3, 5, 7, and 4′.^43^ This group of anthocyanins is red-orange in color and is mainly present in fruits such as strawberries, raspberries, and acerola.^44^ Delphinidin is found in various glycoside forms, and its structural characteristics allow it to act broadly in the human body.^45^ This anthocyanin is present in high concentrations in blue and purple flowers and in foods such as eggplant, grapes, blackcurrants, and some red fruits, with a distribution of 12% delphinidin in fruits and vegetables.^38^ This substance is water-soluble, highly stable under acidic conditions, and unstable when subjected to alkaline pH. For example, gallic acid is a degradation product.^46^

**Figure 6:**
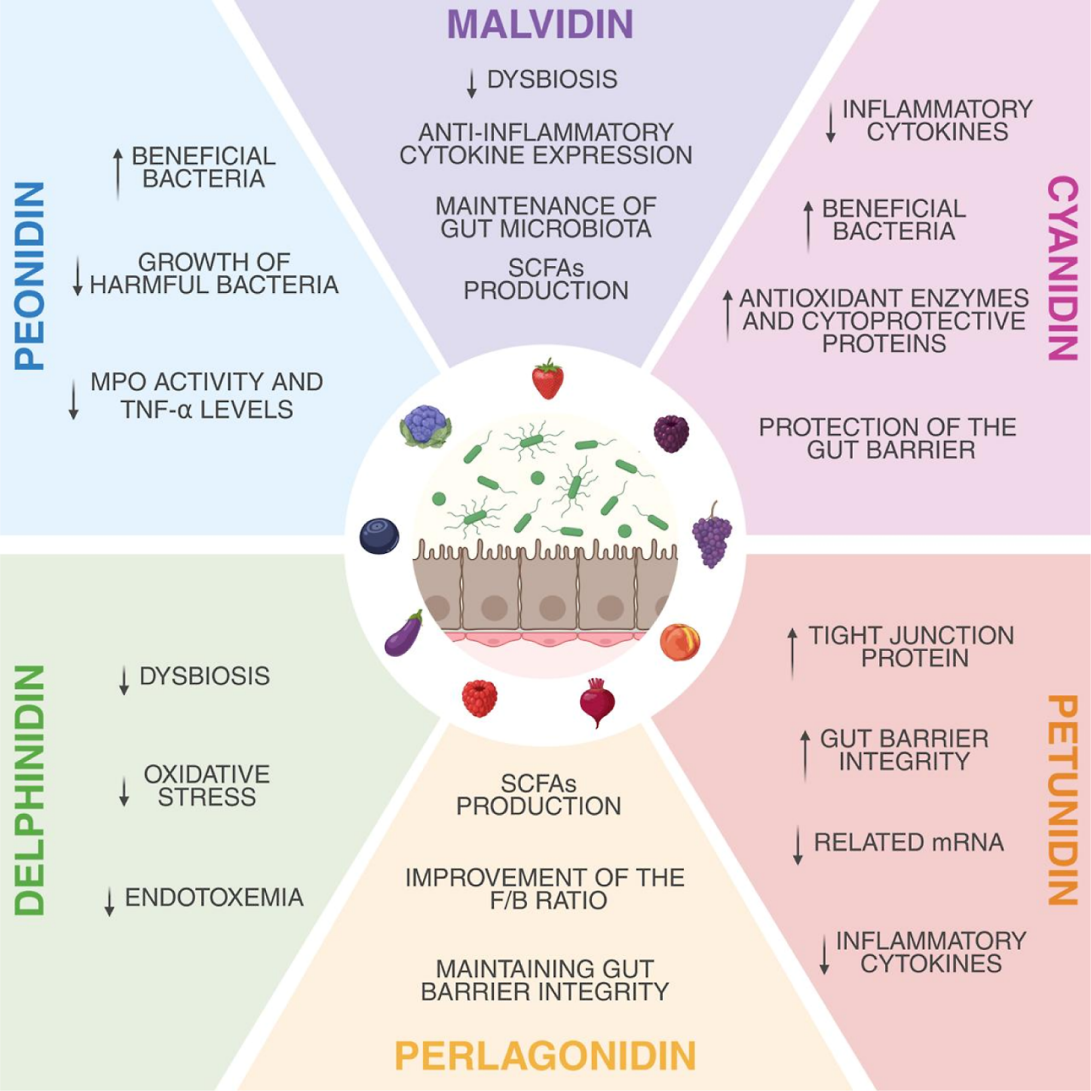
Summary of the principal biological activities of anthocyanidins related to intestinal permeability, highlighting their mechanisms of action. The schematic illustrates how anthocyanidins modulate epithelial barrier integrity through antioxidant, anti-inflammatory, and tight junction-preserving effects. These actions collectively contribute to the maintenance of mucosal homeostasis and attenuation of barrier dysfunction in inflammatory conditions.^40,42,45,47,50,54,58,63–65^ Created in BioRender. Pereira, Q. (2025) https://BioRender.com/542v716.

Table 1 shows the main findings regarding the pharmacological activity of malvidin and its effects on the intestinal microbiota. Only a small portion of malvidin is absorbed by the small intestine. However, a large portion of the tumor undergoes degradation in the colon. The mechanisms by which malvidin exerts its effects on intestinal homeostasis have not yet been fully described; however, it is known that this molecule has high pharmacological potential. In three articles, it was observed that there was a reduction in dysbiosis and promotion of the maintenance of the intestinal microbiota, mainly by an increase in beneficial bacteria and reduction of pathological bacteria.

**Table 1:**
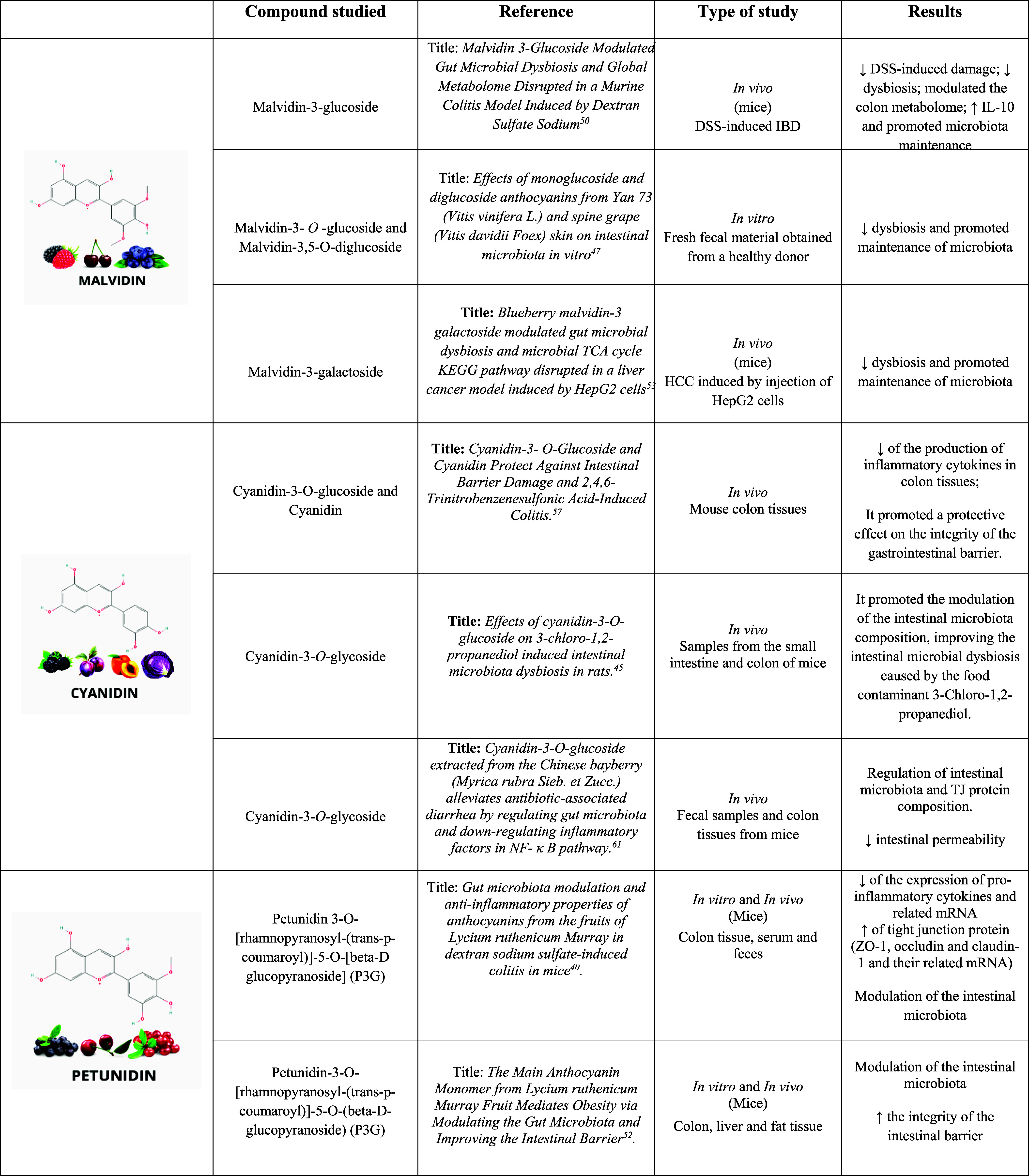

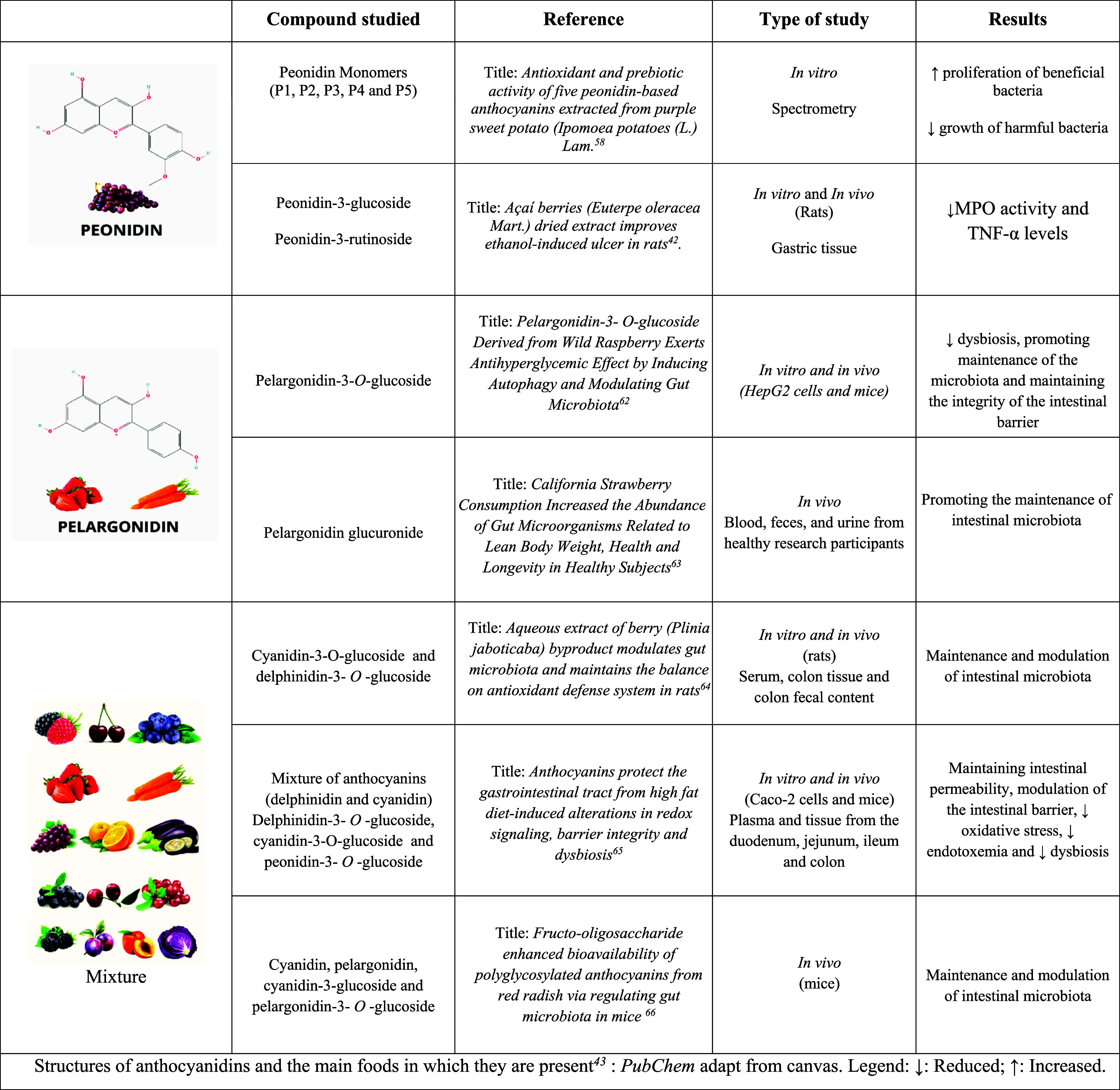
Main Studies Related to Anthocyanins and the Intestinal Microbiota^61,62,66^

In a study carried out with malvidin-3-glucoside, the structure, function, and metabolism of the intestinal microbiota were analyzed after supplementation with malvidin to elucidate the mechanisms underlying the improvement of intestinal dysbiosis in a model of colitis induced by dextran sodium sulfate (DSS). Under physiological conditions, *Firmicutes* and *Bacteroidetes phyla* represent approximately 90% of the fecal bacterial community in adults. A high *Firmicutes*/*Bacteroidetes* (F/B) ratio is generally associated with dysbiosis of the intestinal microbiome, as well as related to a high-fat diet and metabolic diseases.^47^ Thus, an increase in the F/B ratio represents an increase in the disproportionality between the presence of the two phyla described above. Thus, an increase in the *Firmicutes phylum* and a reduction in the *B. phylum* lead to changes in metabolism, insulin resistance, and greater absorption of nondigestible polysaccharides.^48^ Malvidin-3-glucoside improved the F/B ratio, which was related to the increased abundance of the *Clostridia* class, which are considered as butyrate-producing bacteria, a substance that contributes to the restoration of this ratio. Furthermore, after the ingestion of malvidin-3-glucoside, there was a reduction in the damage to the intestinal tissue induced by DSS, decreased inflammatory activity through the improvement of dilation of the intestinal crypts, improvement of intestinal ulceration, and reduction in the thickness of the smooth muscle of the colon, which was associated with an increased expression of the anti-inflammatory cytokine IL-10 in the colon mucosa. In addition, rectal administration of adenoviral vectors containing IL-10 genes in mice with DSS-induced IBD reduced the levels of pro-inflammatory cytokines such as IL-6 and IL-1β. The expression of IL-10 is considered a characteristic of M2 macrophages; thus, malvidin could induce the polarization of M2 macrophages in the colon mucosa.^49,50^ Another important result found with malvidin-3-glucoside was its activity in reducing LPS synthesis and oxidative phosphorylation, decreasing the presence of bacteria that favor inflammation and oxidative stress, which play a significant role in intestinal permeability. In addition, several metabolites associated with colitis symptoms, such as 4-hydroxyphenyl acetate and lipids (ceramides, sphingosines, and sphingosines), are inflammatory mediators that aggravate inflammatory activity and increase intestinal permeability.^50,51^ Operational taxonomic units (OTU) were used to demonstrate the increase or decrease in bacteria after supplementation with malvidin-3-glucoside. Thus, a reduction in *Ruminococcus gnavus* was demonstrated, which is related to the increase in IBD activity and greater abundance of the genera *Clostridium* and *Bacteroides ovatus*.^52^ In the same study, results showed that the action of malvidin on the global metabolome was also analyzed through the quantification of enzymes from the Kyoto Encyclopedia of Genes and Genomes (KEGG) and their metabolites, demonstrating an improvement in the profile of long- and medium-chain fatty acids; the metabolites originating from malvidin-3-glucoside, such as gallic and syringic acids, increased the growth of *Bifidobacterium*, *Lactobacillus*, and *Enterococcus*.

In a study of the molecules malvidin-3-*O*-glucoside and malvidin-3,5-*O*-diglycosidic, three main pharmacological mechanisms were described: an increase in the synthesis of short-chain fatty acids, reduction of intestinal pH, and/or damage to the morphology of bacterial cells through the analysis of the fresh fecal material from a 24-year-old donor who did not have intestinal disease and did not undergo antibiotic treatment in the 6 months prior to the donation. In this study, it was also possible to compare the impact of structural differences between glycosidic and diglycosidic malvidins on intestinal dysbiosis.^47^

Intestinal pH is an indicator of bacterial fermentation and is influenced by the metabolites produced by malvidin. Furthermore, this reduction can inhibit the presence of pathogenic bacteria. Malvidin-3-*O*-glucoside promoted a reduction in pH, unlike malvidin-3,5-*O*-diglucoside, and this difference was explained by the greater stability of diglycosidic acid under physiological conditions, which reduces the production of acidic metabolites, implying a lower capacity to reduce pH. Both molecules increased the production of short-chain fatty acids (SCFAs); however, malvidin-3,5-*O*-diglycosidic supplementation had a greater capacity to increase SCFAs. This result can be explained by the release of two glucose molecules, which can be used directly as substrates for bacterial metabolism to produce more SCFAs, which are the main products of intestinal microbial activity, after the fermentation of dietary fibers, providing energy to the colonic epithelium. SCFAs can act against intestinal pathogens owing to the acidification of bacterial cells and the consequent interruption of their cellular functions, and this effect can be improved in synergy with pH reduction. Thus, malvidin-3-*O*-glucoside increased the production of propionic and *n*-butyric acids, efficiently promoting the alpha diversity of the intestinal microbiota, whereas malvidin-3,5-*O*-diglucoside induced higher concentrations of acetic, propionic, *n*-butyric, *n*-valeric, *i*-valeric, and *i*-butyric acids, resulting in greater bacterial diversity.^47^ Furthermore, it was shown that malvidin-3,5-*O*-diglycoside may have reduced intracellular pH and interrupted the biological cellular activities of *Escherichia shigella*, suppressing the proliferation of this pathogen, in addition to interfering with the integrity of the cell wall of this bacterium.^47^ Both malvidin-3-*O*-glucoside and malvidin-3,5*-O*-diglycoside significantly increased the populations of *Actinobacteria*, *Proteobacteria Bifidobacterium*, *Prevotella*, and *Faecalis bacterium*, but only malvidin-3-*O*-glucoside reduced the F/B ratio.

In the malvidin-3-galactoside study, fecal samples from mice with hepatocellular carcinoma (HCC) were collected to analyze the gut microbiota and KEGG pathway of TCA microbial cycle metabolism after administration of high-dose malvidin (HM3G) and low-dose malvidin (LM3G).^34,53^ At both doses, intestinal microbiota composition and diversity improved, with a reduction in pathogenic bacteria; specifically, the decline of Bacteroides was controlled, while *Clostridial* taxa, including *Oscillospira* and *Ruminococcus*, increased proportionally, and the proliferation of the pathogenic class *Erysipelotrichi* was reduced.

Malvidin-3-galactoside was able to regulate the KEGG pathway of the microbial TCA cycle, improve the expression of key proteins, and regulate microbial carbohydrate metabolism and is considered to have the potential to modulate the intestinal microbiota, as well as for the treatment of HCC.^53^

Cyanidin-3-*O*-glucoside (C3G) has been associated with an increase in *Bacteroides species*, which are known for their beneficial effects and ability to reduce harmful bacteria, such as *Enterococcus* and *Clostridium*. These bacteria are considered harmful when present in excess in the intestine as they cause inflammation and other health problems.^54,55^ Another interesting finding was that C3G may contribute to restoring the homeostasis of the gut microbiota. For example, it was observed that C3G is related to the relative abundance of a bacterium called *Lachnoclostridium*, which produces short-chain fatty acids such as propionate and butyrate. These fatty acids play an important role in gut health, as they promote the proliferation and differentiation of intestinal cells. In addition to protecting the intestinal epithelial barrier,^54^ C3G metabolites can exert protective effects in mouse models of colitis by restoring the abundance of certain beneficial bacteria such as *Lactobacillus* and reducing the population of pathogenic bacteria such as *E. coli*, *Staphylococcus aureus*, and *Pseudomonas aeruginosa*. Thus, C3G metabolites have also been investigated in relation to intestinal health.^56^

In summary, studies suggest that anthocyanins, such as C3G, may have positive effects on the gut microbiota by promoting the growth of beneficial bacteria and reducing the population of harmful bacteria. Furthermore, anthocyanin metabolites play an important role in the gut health. However, further research is needed to fully understand the mechanisms involved and explore the therapeutic potential of anthocyanins in promoting gut health.^39^

In line with the data presented in Table 1, which presents the main findings related to the therapeutic activity of cyanidins. Gan et al.^57^ demonstrated in their study that cyanidins have potential anti-inflammatory activities, enabling their use as new therapeutic options to reduce the risk of chronic diseases such as IBD. In this study, Gan^57^ stimulated colitis induced by 2,4,6-trinitrobenzenesulfonic acid (TNBS), with the experimental groups receiving 200 IL of C3G and cyanidin (Cy) 12 h before TNBS injection and on the subsequent 3 days, and showed that there was a significant improvement in clinical symptoms and histological damage caused by inflammation induced by TNBS through the suppression of the synthesis of inflammatory cytokines, such as TNF-α and interleukins (IL-1β, IL-6, and IFN-c). In addition, cyanidins promoted a protective effect on the intestinal barrier in a monolayer of Caco-2 cells due to an improvement in transepithelial electrical resistance. In a study conducted by Chen et al.,^45^ C3G not only improved dysbiosis but also protected against damage to the intestinal mucosa caused by the food contaminant 3-Chloro-1,2-propanediol (3-MCPD). In their study, we used highly purified C3G extracted from black soybean hulls in different groups of rats (*n* = 40) randomly divided into five groups of eight rats. The experimental groups administered a dose of 20 mg/kg bw per day of 3-MCPD, and the 2 intervention groups received two different doses, 500 mg/kg diet and 1000 mg/kg diet, of an additive diet of 3-MCPD and C3G. After 8 weeks of treatment, the samples were analyzed. Wang et al.,^54^ in their study exploring the therapeutic effect of Chinese Bayberry on antibiotic-associated diarrhea (AAD), revealed that treatment with C3G promotes the richness and diversity of the intestinal microbiota, decreasing the bacterial genera *Enterococcus* and *Clostridium senus* stricto 1. In addition, there was an up-regulation in the expression of intestinal TJ proteins claudin-1 and ZO-1. According to a study by Wang,^54^ the experiment was performed using male mice randomly distributed into different groups and treated with and without C3G. On the eighth day, the mice were euthanized, and specimens of their colons were removed for analysis. There was a significant increase in the abundance of beneficial bacteria and the inhibition of harmful bacteria. Furthermore, the anti-inflammatory activity of C3G has been shown to be effective in restoring intestinal TJ proteins and, consequently, in reducing intestinal permeability. Given the above scenario, various studies have demonstrated several benefits to human health of cyanidin supplementation through its anti-inflammatory activities and modulation of the intestinal microbiota. Thus, the use of anthocyanin as a potential preventive agent or supplemental medication may be a promising therapeutic option for the treatment of chronic diseases and conditions related to inflammation and intestinal health.

In studies conducted by Peng and Liu, petunidins, more specifically petunidin 3-*O*-[rhamnopyranosyl-(*trans*-*p*-coumaroyl)]-5-*O*-[beta-D glucopyranoside] (P3G), have been shown to be important in the modulation of intestinal microbiota as well as in their anti-inflammatory activities. In the first study, presented in Table 1, Peng et al.^40^ sought to evaluate the therapeutic effects of petunidin on dextran sodium sulfate (DSS)-induced colitis in mice. Mice were divided into DSS, P3G-DSS, and ACN-DSS groups, which were used to induce intestinal inflammation in the experimental models. The mice received DSS solution at a concentration of 1.5% in drinking water on days 1–8. These substances were then administered to evaluate their effects on the DSS-induced intestinal inflammation. Throughout the experimental period, daily measurements of the body weight of the mice were taken, and fecal samples were collected for analysis of solid fecal weight. In addition, the food consumption by mice was recorded daily. The severity of inflammatory bowel disease (IBD) was assessed using disease activity index (DAI). This index was calculated based on three parameters: body weight loss, presence of diarrhea, and presence of blood in the stool. Each parameter was assigned a score based on the severity, and these scores were summed to obtain the final DAI value. This methodology allowed us to evaluate the effect of treatment with P3G and ACN on the activity of IBD induced by DSS in mice, providing information about the efficacy of these substances as possible therapies for inflammatory bowel diseases in humans.

It was found that both treatments with P3G and ACN showed a decrease in the expression of pro-inflammatory cytokines (TNF-α, IL-6, IL-1β, and IFN-γ), in addition to modulating the intestinal microbiota and increasing the integrity of the intestinal barrier.

In the second study,^52^ the methodology used by Liu et al. involved the formation of three groups of mice: mice fed a normal diet, in which 10% of the energy came from fat; those fed a high-fat diet, in which 60% of the energy came from fat; and mice that were fed the high-fat diet described above but also received oral administration of P3G at a dose of 100 mg/kg of body weight. The NC and high-fat diet groups received sterile water daily by gavage, simultaneously with feeding. This water administration was intended to provide hydration to the mice. In the 11th week of the experiment, a fasting blood glucose test and an oral glucose tolerance test (OGTT) were performed for all groups. These tests were performed to evaluate glucose metabolism and the response of mice to glucose intake. At the end of the experiment, all mice were fasted for 12 h and then sacrificed. Plasma and tissues, including the fat, liver, and colon, were collected for biochemical analysis. These biochemical analyses allowed for the evaluation of parameters related to metabolism, inflammation, and other variables relevant to the study. This methodology allowed us to investigate the effects of a high-fat diet and P3G administration in mice, providing information about glucose metabolism, inflammatory responses, and other biochemical changes in the tissues studied. Thus, P3G has a potential mechanism of action in both regulating the intestinal microbiota and protecting the intestinal barrier.

In studies related to peonidin, the authors demonstrated that it exerts important anti-inflammatory and antioxidant functions in addition to modulating intestinal microbiota. The first such study was conducted by Sun et al.^58^ who evaluated the scavenging capacities of 1,1-diphenyl-2-picrylhydrazyl (DPPH) radicals and superoxide anions as well as the total reducing power of peonidin-based anthocyanins. Spectral analysis and concentration tests were performed to determine the antioxidant activities of different anthocyanins. The study showed that peonidin-based anthocyanins may not only be good antioxidant agents but also potential natural probiotic sources that can enhance the proliferation of *Bifidobacteria* strains (*B. infants*, *B. adolescents*, and *B. bifidum*) and *Lactobacillus acidophilus*, as well as inhibit the growth of intestinal pathogens *S. aureus* and *Salmonella typhimurium*. In the second study, Cury et al.^42^ divided rats into groups according to the following treatments: omeprazole (30 mg/kg), water (10 mL/kg), dry extract of açaí berries (DAE) (30–300 mg/kg), and euthanized after the administration of 98% ethanol (5 mL/kg). The stomachs of the rats were opened, and the ulcer area was measured. The results of this study demonstrated that the extract showed radical-scavenging activity in vitro, maintaining the oxidative balance of the gastric mucosa, and gastroprotective effects in vivo, reducing ulceration (Table 1).

Delphinidin is a key red-orange anthocyanidin that is abundant in strawberries, raspberries, and blueberries. It has also been quantified in a variety of other fruits, including cranberries, sweet cherries, red and pink currants, pomegranates, peaches, plums, and blackberries, and occurs in certain vegetables and pulses, such as red radish, beetroot, and common beans.^59,60^ The first study analyzed the effects of pelargonidin-3-*O*-glucoside (Pg3G), derived from wild raspberry, in reducing hyperglycemia in hepatocytes and its underlying mechanisms of action in autophagy and modulation of the intestinal microbiota. To identify the fecal microbiota, 16S rRNA sequencing of the cecal content of mice was performed, and fecal SCFA levels were obtained through analysis of the fecal content. First, a procedure was performed to purify anthocyanins, and three main compounds were identified: cyanidin-3-*O*-glucoside, Pg3G, and pelargonidin-3-*O*-rutinoside. Pg3G was considered the predominant anthocyanin in wild raspberry, representing 91.76% of the total anthocyanin content in the fruit, and it was subsequently isolated. In an in vitro study, HepG2 cells were induced by high glucose and fat (HG + HF) to represent the condition of a diabetic organism. In addition, the cells were labeled to evaluate the glucose uptake. After treatment with Pg3G at doses of 5, 10, and 20 μg/mL, it was able to reverse the reduction in glucose uptake induced by HG + HF and had an effect on liver function and reduced hyperglycemia. It is believed that Pg3G can promote glucose uptake by inducing autophagy, a process involved in glucose and lipid homeostasis, since Pg3G increased the expression of LC3B protein in HepG2 cells when administered at concentrations of 2.5, 5, 10, and 20 μg/mL, which is an important marker of autophagy. Given that Pg3G influences autophagy, a relationship between anthocyanin and transcription factor EB (TFEB), which is a regulator of autophagy and lysosomal biogenesis, was hypothesized. It was proven that Pg3G restored the expression of the nuclear protein TFEB, resulting in the accumulation of lysosomes in cells, which could lead to the activation of TFEB. Similar results in vivo were observed, by increasing the expression of the hepatic proteins LC3B and TFEB in diabetic mice and the accumulation of autophagosomes in the liver tissue, suggesting an improvement in glucose homeostasis and insulin sensitivity.

The intestinal microbiota of mice that received Pg3G were notably distinct from that of mice that did not receive this anthocyanin. Through the analysis, it was possible to identify the decrease and alteration of the diversity and abundance of some bacterial species, such as an increase in the abundance of *Bacteroidetes* and a reduction in the abundance of *Firmicutes*. This increase in the B/F ratio was associated with the reduction of plasma glucose and insulin resistance, in addition to the greater abundance of *Bacteroidia*, *Bacteroidales*, *Prevotella*, and *Prevotellaceae*, with the modulation of the abundance of *Prevotella* being associated with the improvement of glucose metabolism.

Furthermore, an increase in SCFAs derived from fermentation by the intestinal microbiota, including acetate, propionate, butyrate, isobutyrate, and valeric acid, was observed, which may also be associated with an increase in the *Prevotella genus*, which is considered a producer of SCFAs.

Another important result was that Pg3G was able to maintain the integrity and improve the function of the intestinal barrier in several ways: due to the increase in the amount of total SCFA, since the reduction of these acids is related to the weakening of tight junctions and increased intestinal permeability; due to the increased expression of occludins, tight junction-1, and bacterial peptides (phospholipase A2 group II and lysosome-1), which strengthens the intestinal barrier and reduces dysbiosis due to the increased expression of mucin 2, which constitutes the mucus capable of protecting and lubricating the intestinal wall, and due to the activation of Toll-like receptor 2 (TLR2), which regulates tight junction proteins. Finally, it was possible to identify that Pg3G improved glucose tolerance, insulin tolerance, fasting insulin levels, and the homeostatic model assessment index for insulin resistance by performing glucose and insulin tolerance tests after the administration of 150 mg/kg Pg3G to mice. The second article evaluated whether the consumption of strawberry powder (SBP) could alter the intestinal microbiota and intestinal metabolism of cholesterol and bile acids in healthy individuals who consumed a diet low in fiber and polyphenols.

After 4 weeks of SBP consumption, urine and blood samples were collected from the study participants to determine pelargonidin glucuronide, urolithin A glucuronide (UAG), and dimethyl ellagic acid glucuronide (DMEAG), with UAG and DMEAG being two intestinal metabolites of ellagitannins, a class of hydrolyzable tannins. Fecal samples were also collected before, during, and after SBP treatment to quantify cholesterol, bile acids, and SCFA and for bacterial DNA sequencing and subsequent identification of operational taxonomic units (OTUs). Other parameters, such as weight, body composition, and serum total HDL cholesterol and triglyceride levels, were also analyzed. Pelargonidin glucuronide was detected in the serum and urine of all individuals, and metabolites UAG and DMEAG were detected in urine. In the stool samples, no significant changes were identified in the fecal concentration of SCFA over weeks; however, there were interindividual changes in fecal cholesterol and bile acids. SBP did not cause changes in the abundance of intestinal microbiota phyla; however, 24 molar oxidation units (OTUs) were altered considerably. Thus, there was an increase in the abundance of OTUs from the families *Christensenellaceae*, *Bacteroidaceae*, *Bifidobacteriaceae*, and *Verrucomicrobiaceae* and a reduction in the phylum *Alcaligenaceae*/*Sutterella* during treatment. In addition, after 4 weeks of SBP consumption, there was an increase in the *Clostridia* class, but the difference was not significant. After discontinuation of SBP consumption for 2 weeks, we analyzed whether the changes identified in the intestinal microbiota during treatment were maintained. We found that the abundance of *Christensenellaceae*, *Verrucomicrobiaceae*, and *Mogibacteriaceae* was reversed, and the abundance of four OTUs of the *Clostridia* class and one OTU of the *Bacteroidia* class was reduced, whereas two OTUs of each class mentioned above increased. There were no significant changes in the alpha or beta diversity. There were no changes in weight, body composition, or serum levels of high-density lipoprotein cholesterol and triglycerides over the weeks.

The changes considered most important were those that changed in the 2 weeks after discontinuation of SBP consumption, such as the reversed abundance of the *C. Christensenellaceae* family, the *Akkermansia muciniphila* family, and *Mogibacteriaceae family*. Although the results of the study led to the conclusion that SBP contributed to a microbiota composition that was beneficial to health, body weight, and longevity, some biases were identified: the lack of an adequate placebo group since, in the initial project, compounds with prebiotic characteristics were identified in the powder to be ingested by the group; the study design, with a crossover design being identified as more suitable for the development of the research; the “beige diet” that the research participants were instructed to consume may have limited the nutrients necessary for the formation of SCFA and the increase in cholesterol and bile acid metabolizing bacteria; and the calculation of power was based on the primary outcome parameter (fecal microbiota).

Table 1 shows the results of the studies that used more than one type of anthocyanin to evaluate its pharmacological activity. In the first study, Silva-Maia et al. analyzed the aqueous extract of jabuticaba peel (JAE) in vitro, through the identification of its compounds, and in vivo, through the ingestion of JAE in rats. This study aimed to observe modulation of the intestinal microbiota and maintain the balance of the antioxidant defense system. In vitro analysis cyanidin-3-*O*-glucoside and delphinidin-3-*O*-glucoside as the main phenolic compounds present in the extract, with a concentration of 52.53 ± 5.37 mg L^–1^ and 2.29 ± 0.44 mg L^–1^, respectively. JAE was able to modulate the microbiome due to the proliferation of enterobacteria and bifidobacteria, as well as due to the increase in the *Lactobacillus* genus during the 2 weeks of treatment, while the total amount of aerobic bacteria was not affected. Furthermore, there was an increase in the production of fatty acid acetate in the colon of rats treated for 7 weeks, a substance that has the potential to improve glucose tolerance. However, there was no change in the concentration of propionate, butyrate, and short-chain fatty acids (SCFAs).

Finally, antioxidant activity was also analyzed, identifying an increase in serum superoxide dismutase (SOD) activity after JAE ingestion, whereas rats that ingested JAE for 7 weeks showed a reduction in serum catalase (CAT) activity and an increase in its activity in the colon. A study developed by Cremonini et al. analyzed, in vivo, the capacity of a mixture rich in anthocyanins (ACN), with an emphasis on delphinidin and cyanidin, to sustain the integrity of the intestinal monolayer, prevent endotoxemia, and prevent dysbiosis after the exposure of mice to a high-fat diet. Furthermore, the permeability of the Caco-2 cell monolayer was evaluated in vitro, which were incubated with the mixture of ACN, protocatechuic acid (PCA) or with the 3-*O*-glycosides of delphinidin, cyanidin, and peonidin and treated with tumor necrosis factor γ (TNFγ) to promote permeabilization.

As previously mentioned, poor nutrition is one of the factors that alters intestinal permeability and promotes endotoxemia, in addition to causing several other disorders in the body. Thus, mice fed a high-fat diet (HFD) develop obesity, insulin resistance, and altered expression of TJ proteins in the ileum. Mice ingested fluorescein isothiocyanate (FITC)-dextran, a marker for assessing intestinal permeability through paracellular transport. FITC-dextran transport and plasma endotoxin levels were higher in HFD-fed mice that received a HFD, which was characterized by increased intestinal permeability. However, in mice that received ACN, plasma endotoxin levels were similar to those in the control group, which received a low-fat diet, and there was also a decrease in the altered expression of TJ proteins. The plasma levels of glucagon-like peptide 2 (GLP-2) were higher in the groups that received ACN. This peptide is important for regulating glycemic metabolism, especially considering the impact of a high-fat diet on obesity and insulin resistance. Furthermore, the amount and distribution of mucin 2 (MUC2) in the ileum of mice were measured, and a reduction in the substance and its accumulation in the cell calyx were observed in mice that ingested DH, which was avoided in the groups that received the ACN mixture. It is important to highlight that MUC2 is a component of the mucus layer responsible for protecting the intestinal epithelium and constituting the first line of immunological defense.

Regarding the impact of the high-fat diet on the intestinal microbiota, it caused an increase in the F/B ratio (there was an increase in the abundance of *Firmicutes* and a reduction in *Bacteroidetes*) and a reduction in the abundance of *Akkermansia*, after which it was observed that both changes were avoided with the administration of the anthocyanin mixture. Finally, another important mechanism for the protection of the intestinal barrier was observed since the ACN mixture inhibited the positive regulation of NADPH oxidases, an enzyme capable of generating reactive oxygen species, thus reducing oxidative stress and activating redox-sensitive signaling. It also prevented the increase in phosphorylation of NF-κB and ERK1/2, which are involved in the modulation of the structure and function and the phosphorylation of ileal myosin (MLC).

In in vitro study, the ACN mixture prevented an increase in FITC-dextran permeability and reduced transepithelial electrical resistance (TEER) in Caco-2 cells. Furthermore, PCA, cyanidin-3-*O*-glucoside, and the ACN mixture prevented the phosphorylation of NF-κB, ERK1/2, and MLC, and delphinidin-3-*O*-glucoside inhibited only the phosphorylation of NF-κB triggered by TNF-α. PCA, cyanidin-3-*O*-glucoside, and peonidin-3-*O*-glucoside inhibited the upregulation of NADPH oxidases. The third study, conducted by Lia et al., aimed to evaluate the effect of fructooligosaccharide (FOS) on the bioavailability of red radish anthocyanins (ARR) by analyzing anthocyanidins, anthocyanins, and phenolic acids in the serum and cecum of mice, analyzing antioxidant parameters in liver tissue, 16S rRNA sequencing of the colon microbiota of mice, and fecal bacteria transplantation (FMT) in pseudointestinal germ-free mice. After analyzing the red radish extract, 2 anthocyanidins and 17 anthocyanins were identified; among them, pelargonidin-3-diglycoside-5-(malonyl)­glucoside (P3D5MG), pelargonidin-3-(feruloyl)­diglycoside-5-(malonyl)­glucoside (P3FD5MG), and pelargonidin-3-diglycoside-5-glucoside (P3D5G) were considered as anthocyanins with a complex molecular structure. The main anthocyanins were not found in the serum of mice treated with ARR with or without FOS; however, in mice treated with FOS + ARR, after hydrolyzing the samples, greater bioavailability of pelargonidin (P), cyanidin (Cy), and pelargonidin-3-*O*-glucoside (P3G) was identified, whereas there was no difference in serum cyanidin-3-glucoside (C3G) levels in any of the treatments.

The phenolic acids protocatechuic (PA), caffeic (CA), gallic (GA), and *p*-hydroxybenzoic (PHBA) were also analyzed because they are important anthocyanin metabolites generated by the intestinal microbiota. No significant levels of these substances were identified in the original or hydrolyzed serum samples from mice treated with ARR or FOS + ARR.

Individual treatment with ARR resulted in higher total antioxidant activity (T-AOC) and increased superoxide dismutase (SOD) and glutathione peroxidase (GSH-Px) activities, whereas the FOS + ARR combination treatment demonstrated even higher SOD and GSH-Px activity than individual treatments. The improved bioavailability of anthocyanins P, C, and P3G can be correlated with the increased antioxidant activity identified in liver tissue.

The presence of anthocyanins and phenolic acids in the cecum of mice was analyzed to determine the intestinal metabolism of ARR. Unlike the results obtained in the serum, anthocyanins with complex molecular structures were found, P3CD5G, P3FD5G, and P3PD5G, which can be justified because acylated anthocyanins are more difficult to absorb and are kept in the cecum and large intestine for later metabolism, in addition to being difficult to degrade. In mice treated with the FOS + ARR combination, the levels of P and hydrolyzed P glycosides were higher than those in mice treated with ARR alone. Regarding phenolic acids, mice treated with FOS + ARR had higher cecal concentrations of GA and PA, as well as CA and PHBA, but without significant differences between the groups.

Ingestion of the treatment alone or in combination with FOS caused significant changes in the intestinal microbiota. The main phyla found in the colons of mice were *Firmicutes*, *Bacteroidetes*, and *Verrucomicrobiota*. In both treatments, there was an increase in the abundance of *Firmicutes* and a reduction in the abundance of *Bacteroidetes*, which improved the F/B ratio. Finally, FMT was performed to verify the action of the intestinal microbiota on ARR metabolism regulated by FOS. The mice that received the transplant from the FOS + ARR group presented higher serum concentrations of P, P3G, and Cy and higher cecal concentrations of P and C3G than those from the ARR group, indicating that there is an interaction between the microbiota and FOS that favors the bioavailability of ARR.

### Microbiota, Senescence, and Anthocyanins

Aging is intrinsically associated with immunosenescence and inflammation. The inflammatory process resulting from immunosenescence leads to a reduction in serum levels of anti-inflammatory cytokines, such as IL-10, along with a decrease in pro-inflammatory cytokines, such as IL-1β, IL-6, IL-8, and TNF-α. Such changes play crucial roles in the aging of the immune system and its responses.^67^ Recent studies have considered the relationship between senescence and microbial dysbiosis.^68–70^ An important study directly evaluated the microbial composition in murine models of senescence, and intestinal microbial signatures associated with markers of cellular senescence and inflammatory SASP factors were identified. The data revealed that the expressions of p16^Ink4a^, p21^Cip1^, and IL-6 were negatively correlated with the genus *Dorea* in the ileum. In contrast, IL-1β was negatively linked to *Peptococcaceae*, and CXCL1 was positively linked to *Staphylococcus* and *Clostridial* in the same intestinal portion. The abundance of Firmicutes and the reduction in Bacteroides were associated with the expression of CXCL1 in the coccus of these animals. Other markers, such as p16^Ink4a^ and monocyte chemoattractant protein-1 (MCP-1), also showed central connections with several bacterial species in the cecum. However, similar patterns have been observed in the ileum, cecum, and colon of several species. A positive correlation was observed between the panel of inflammatory and senescence markers (including TNF-α) and members of the *Clostridiales* order, *Staphylococcus* spp., and taxa within the *Lachnospiraceae* family across the sampled tissues. In contrast, *Coriobacteriaceae* and *Akkermansia* showed consistent negative correlations with these markers in all tissues examined. These results indicated a robust association between specific microbial signatures and markers of cellular senescence, supporting the hypothesis that gut dysbiosis is linked to and may contribute to the senescence-associated inflammatory phenotype.^69^

Similarly, another study demonstrated that intestinal tissue developed characteristics of cellular senescence, as evidenced by the increased expression of senescence markers, such as p16^Ink4a^, p21^Cip1^, and SA-β-gal in WT mice and Ercc1-/Δ-accelerated aging murine models.^71^ Additionally, an age-dependent increase in DNA damage has been described, in addition to aspects associated with cellular senescence (p53/p21WAF1), activation of SASP regulators (NF-κB, p38MAPK, and Cox-2), and metabolic stress in the intestinal tissue of aged mice, indicating an increased susceptibility to genotoxic stress in aging.^72^ Complementarily, it was shown that intestinal epithelial stem cells derived from aged mice presented increased mRNA levels of genes associated with cellular senescence and oxidative stress,^73^ while intestinal epithelial organoids from aged animals also showed consistent upregulation of senescence markers, such as SA-β-gal and p21 activity, compared to organoids derived from younger organisms.^74^ Furthermore, another study showed that radiation exposure induces premature cellular senescence and the SASP phenotype in intestinal stem cells in vivo.^75^ Thus, it is evident that the intestinal epithelium and stem cells express signatures of age-dependent cellular senescence, contributing to known functional alterations and disruption of gastrointestinal homeostasis. Additionally, chronic secretion of inflammatory SASP factors by senescent intestinal cells can promote an inflammatory environment and/or oncogenic transformation, which may result in impairment of intestinal permeability, immune activation, and composition of the intestinal microbiome.^72,75^ A study reported the role of the senescent environment of colonic tissue, including the SASP, in the pathogenesis of colorectal cancer, an age-related disease. The data indicated an increased number of senescent stromal cells in the colon stroma associated with increased secretion of GD15, a SASP factor secreted by senescent colon cells involved in cell proliferation, migration, and invasion in colon adenoma.^76^ Although cellular senescence is undesirable in the context of aging, pro-senescence strategies are often considered favorable for preventing cancer cell proliferation.^77^

The gut microbiome uses dietary fuel to synthesize a series of bioactive metabolites, such as SCFAs, phenols, neurotransmitters, HPA hormones, endotoxins, and ammonia, through processes such as microbial fermentation.^78,79^ These metabolites can reach the circulation and affect the functioning of organs and different organic systems, factors that lead the gut microbiome to be considered a complex virtual endocrine organ.^80–82^ Although senescence is subject to multiple factors, the natural development of this mechanism can be regulated by the cellular capacity to respond to stress and oxidative damage mediated by ROS.^83^

Increased oxidative stress throughout an animal’s life can directly increase the accumulation of senescent cells, and the application of antioxidants can attenuate cellular senescence both in vitro and in vivo.^84–86^ Therefore, studies have indicated that the development and accumulation of senescent cells can be attenuated or even delayed by increasing the potency of the cellular stress response and redox balance through the neutralization of oxidative stress and inflammatory events. In this regard, it is known that several metabolites from the gut microbiome and probiotic bacteria or microbiome-fermented dietary phytomolecules exert strong anti-inflammatory and antioxidant attributes that may be useful in preventing pro-inflammatory and tumorigenic environments associated with senescence.^87–90^ Bioactive compounds such as polyphenols are phytomolecules that are poorly absorbed in the small intestine and are fermented by the colonic microbiota to produce several simple molecules that can have several beneficial biological effects at the tissue and systemic levels.^87,91,92^ Many studies have shown that anthocyanins exert antiaging effects. A study in which aged rats were fed anthocyanin from purple sweet potato demonstrated that this polyphenol significantly reduced the serum levels of malondialdehyde (MDA) and improved the activities of superoxide dismutase (SOD) and glutathione peroxidase (GSH-PX), suggesting that the intake of low doses of anthocyanins could achieve the same effect as the same equivalent amount of vitamin C, indicating that anthocyanin from purple sweet potato may play a role in delaying aging by improving antioxidant activity.^93^ Another study showed that Cyanidin-3-*O*-glucoside (Cy-3-glu) and pelargonidin-3-glucoside (Pg-3-glu) treatments significantly inhibited β-galactosidase activity in the aging process of human retinal pigment epithelium (RPE) cells induced by visible light irradiation and played a protective role against antiaging.^55^ A study reported that *Ribes meyeri* anthocyanins promote neural stem cell proliferation and attenuate the cellular senescence phenotype by reducing EROS production and senescence-associated p16^Ink4a^ expression, enhancing DNA synthesis, and prolonging telomere length.^94^ A separate report indicated that anthocyanins preserve redox homeostasis in the plasma and liver and concomitantly reduce hepatic proinflammatory cytokines, including IL-1, IL-6, and TNF-α. At the same time, the decrease in the expression levels of sensor targets (ATM and ATR), intermediates (H2AX and γ-H2AX), and effectors (Chk1, Chk2, p53, and p-p53) in the DNA damage signaling pathway indicates that anthocyanins can delay aging, culminating in the inhibition of DNA damage.^95^ Furthermore, it has been postulated that there may be a relationship between the consumption of foods rich in anthocyanins and the composition of the intestinal microbiome, which includes enrichment of microbial diversity in general, as well as increased production of specific microbial metabolites.^56,96^ The effect of anthocyanins on microbial diversity in the intestine may be particularly beneficial in reducing the risk of developing CVD and colorectal cancer, as these diseases have been associated with microbiome imbalance.^97–99^ Finally, modifications in the intestinal microbiota have been partially linked to the neuroprotective properties of anthocyanin-rich blackberry extract in Wistar rats fed a high-fat diet.^100^ Anthocyanins have been shown to be potentially beneficial in senescence-related events through different mechanisms. They are particularly important in modulating the intestinal microbiota, resulting in local and systemic effects and thus preventing the development of age-related chronic diseases associated with age. A summary of the main findings is shown in Figure 7.

**Figure 7:**
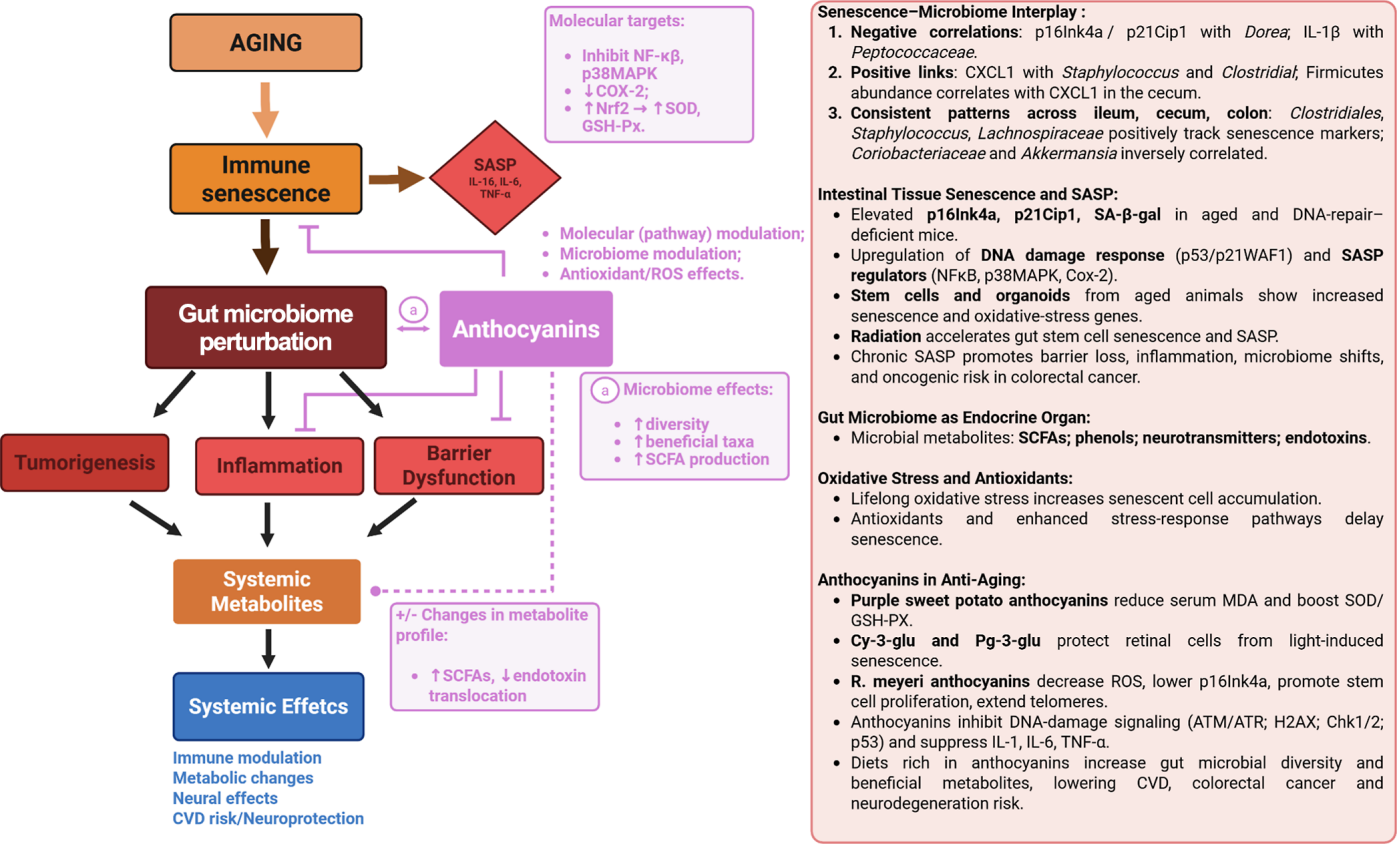
Schematic of the senescence–microbiome interplay in aging showing how immune senescence and SASP link to specific gut bacteria across intestinal sites, drive inflammation and barrier dysfunction, and increase oncogenic risk; anthocyanins counteract oxidative stress, modulate microbiota, and reduce senescence-associated outcomes. SASP represents the senescence-associated secretory phenotype and highlights key cytokines IL-1β, IL-6, and TNF-α as proinflammatory mediators released by senescent cells. Anthocyanins/anthocyanidins act at molecular (NF-κB/MAPK/Nrf2), cellular (SASP, barrier integrity), and microbial (diversity, SCFA production) levels to suppress inflammation, modulate gut microbiota, and alter systemic metabolite profiles, thereby reducing age-associated systemic effects. Arrow styles: flat-headed = inhibition/suppression; dashed = modulation/indirect; and solid = causation/transport. Created in BioRender. Pereira, Q. (2025) https://BioRender.com/542v716.

## Concluding Remarks and Future Perspectives

Current evidence indicates that anthocyanins effectively modulate gut microbial homeostasis by correcting dysbiosis, enhancing the production of short-chain fatty acids and other beneficial metabolites, and protecting against inflammatory bowel disease. Moreover, the reciprocal interplay between cellular senescence and the microbiome underlies intestinal aging, which is marked by DNA damage responses, SASP secretion, barrier dysfunction, and inflammation. Antioxidants, particularly anthocyanins, such as cyanidin-3-*O*-glucoside, attenuate oxidative stress, stabilize redox balance, suppress senescence signaling, and enrich microbial diversity. Future studies should integrate senolytic agents, dietary antioxidants, and targeted microbiome modulation, advancing from murine models to human trials, to elucidate the pharmacological mechanisms and harness the senescence–microbiome axis for disease prevention and healthy aging.

The conclusions of this review are constrained by the predominant use of in vivo models and the limited number of studies on isolated anthocyanins. Rodent studies, which are invaluable for mechanistic exploration, differ substantially from those on human physiology in terms of digestive processes, metabolic rates, and gut microbial composition. Consequently, dose–response relationships and efficacy data derived from these models may not be directly translatable to clinical contexts. To bridge these gaps, we recommend the following research priorities: (a) translate preclinical findings into well-designed human trials that include standardized dosing regimens, rigorous randomization, and extended monitoring of safety and efficacy; (b) combine dietary anthocyanins with senolytic agents to evaluate synergistic effects on senescent cell clearance, microbiome restoration, and intestinal barrier function; (c) establish and validate consensus protocols for anthocyanin extraction, quantification, and molecular characterization (e.g., HPLC–MS/MS and NMR) to ensure reproducibility; (d) investigate anthocyanin pharmacokinetics and bioavailability in humans, including the role of gut microbial metabolism in generating active metabolites; and (e) leverage multiomics approaches (metagenomics, metabolomics, and transcriptomics) to dissect the senescence–microbiome axis and identify key pharmacological targets. Addressing these priorities will clarify the mechanistic basis of anthocyanin action, enable evidence-based dietary recommendations, and advance the development of anthocyanin-based therapeutics for inflammatory bowel disease and healthy gut aging.

## Abbreviations

μg: microgram
3-MCPD: 3-Chloro-1,2-propanediol
AAD: antibiotic-associated diarrhea
ACN: anthocyanins
ARR: red radish anthocyanins
B/F: *Bacteroidetes*/*Firmicutes*
bw: body weight
C3G: cyanidin-3-*O*-glucoside
CAT: catalase
CD: Crohn’s disease
CIDs: chronic inflammatory diseases
Cox-2: cyclooxygenase-2
Cy: cyanidin
DAE: dry extract of açaí berries
DAI: disease activity index
DMEAG: dimethyl ellagic acid glucuronide
DNA: DNA
DSS: dextran sodium sulfate
DPPH: 1,1-diphenyl-2-picrylhydrazyl
e.g.: *exempli gratia*
ERK: extracellular signal-regulated kinase
EROS: reactive oxygen species
F/B: *Firmicutes*/*Bacteroidetes*
FMT: fecal bacteria transplantation
FOS: fructooligosaccharide
GIT: gastrointestinal tract
GLP-2: glucagon-like peptide 2
GSH-Px: glutathione peroxidase
HCC: hepatocellular carcinoma
HDL: high-density lipid
HFD: high-fat diet
HG + HF: high glucose and fat
HM3G: high-dose malvidin
HP2: haptoglobin 2
HPA: hypothalamic-pituitary-adrenal
IBD: inflammatory bowel diseases
IFN: interferon
IL: interleukin
JAE: extract of jabuticaba peel
KEGG: Kyoto Encyclopedia of Genes and Genomes
kg: kilogram
L: liter
LC3B: microtubule-associated protein 1A/1B-light chain 3B
LM3G: low-dose malvidin
LPS: lipopolysaccharides
MCP-1: monocyte chemoattractant protein-1
MDA: malondialdehyde
MeSH: Medical Subject Headings
mg: milligram
mL: milliliter
MLC: myosin
MPO: myeloperoxidase
MUC2: mucin 2
NADPH: nicotinamide adenine dinucleotide phosphate
NF-κB: nuclear factor kappa-B
nm: nanometers
OGTT: oral glucose tolerance test
OTU: operational taxonomic units
P: pelargonidin
P3D5G: pelargonidin-3-diglycoside-5-glucoside
P3D5MG: pelargonidin-3-diglycoside-5-(malonyl)­glucoside
P3FD5MG: pelargonidin-3-(feruloyl)­diglycoside-5-(malonyl)­glucoside
P3G: petunidin 3-*O*-[rhamnopyranosyl-(*trans*-*p*-coumaroyl)]-5-*O*-[beta-D glucopyranoside]
PCA: protocatechuic acid
Pg3G: pelargonidin-3-*O*-glucoside
pH: hydrogen potential
PHBA: *p*-hydroxybenzoic
RPE: retinal pigment epithelium
rRNA: rRNA
SA-β-gal: senescence-associated beta-galactosidase
SASP: senescence-associated secretory phenotype
SBP: strawberry powder
SCFAs: short-chain fatty acids
SOD: superoxide dismutase
T-AOC: total antioxidant activity
TCA: tricarboxylic acid
TEER: transepithelial electrical resistance
TFEB: transcription factor EB
TJ: tight Junction
TLR2: toll-like receptor 2
TNBS: trinitrobenzenesulfonic acid
TNF: tumor necrosis factor
UAG: glucuronide
UC: ulcerative colitis
ZO: zonulin
WT: wild type.
